# ZEB1‐mediated vasculogenic mimicry formation associates with epithelial–mesenchymal transition and cancer stem cell phenotypes in prostate cancer

**DOI:** 10.1111/jcmm.13637

**Published:** 2018-05-12

**Authors:** Hua Wang, Bin Huang, Bai Mou Li, Kai Yuan Cao, Chen Qiang Mo, Shuang Jian Jiang, Jin Cheng Pan, Zong Ren Wang, Huan Yi Lin, Dao Hu Wang, Shao Peng Qiu

**Affiliations:** ^1^ Department of Urology The First Affiliated Hospital of Sun Yat‐Sen University Guangzhou China; ^2^ Research Center for Clinical Laboratory Standard Zhongshan School of Medicine Sun Yat‐sen University Guangzhou China; ^3^ Department of Urology Hui Ya hospital of The First Affiliated Hospital Sun Yat‐Sen University Guangzhou China

**Keywords:** CSC, EMT, prostate cancer, vasculogenic mimicry, ZEB1

## Abstract

The zinc finger E‐box‐binding homeobox 1 (ZEB1) induced the epithelial–mesenchymal transition (EMT) and altered ZEB1 expression could lead to aggressive and cancer stem cell (CSC) phenotypes in various cancers. Tissue specimens from 96 prostate cancer patients were collected for immunohistochemistry and CD34/periodic acid–Schiff double staining. Prostate cancer cells were subjected to ZEB1 knockdown or overexpression and assessment of the effects on vasculogenic mimicry formation in vitro and in vivo. The underlying molecular events of ZEB1‐induced vasculogenic mimicry formation in prostate cancer were then explored. The data showed that the presence of VM and high ZEB1 expression was associated with higher Gleason score, TNM stage, and lymph node and distant metastases as well as with the expression of vimentin and CD133 in prostate cancer tissues. Furthermore, ZEB1 was required for VM formation and altered expression of EMT‐related and CSC‐associated proteins in prostate cancer cells in vitro and in vivo. ZEB1 also facilitated tumour cell migration, invasion and clonogenicity. In addition, the effects of ZEB1 in prostate cancer cells were mediated by Src signalling; that is PP2, a specific inhibitor of the Src signalling, dose dependently reduced the p‐Src^527^ level but not p‐Src^416^ level, while ZEB1 knockdown also down‐regulated the level of p‐Src^527^ in PC3 and DU‐145 cells. PP2 treatment also significantly reduced the expression of VE‐cadherin, vimentin and CD133 in these prostate cancer cells. Src signalling mediated the effects of ZEB1 on VM formation and gene expression.

## 1 BACKGROUND

Prostate cancer is a significant health problem and growing challenge to Chinese men due to the population ageing.^1^ Globally, PCa is usually diagnosed at advanced stages of disease, which are more likely to metastasize to other organs and have high mortality rates.^2^ This is because treatment options for advanced PCa are limited; chemoradiation therapy and hormone therapy have limited effectiveness.^3^ To date, the exact aetiology of PCa remains to be defined, and primary risk factors include obesity, old age, race and family history.^4^, ^5^, ^6^, ^7^ The development of PCa is usually related to prostatic intraepithelial neoplasia^8^ due to the silencing of tumour suppressor genes and/or activation of oncogenes.^9^, ^10^, ^11^ However, further research on PCa pathogenesis and molecular mechanisms could help identify biomarkers for early cancer detection and prediction of treatment responses and prognosis, as well as novel treatment strategies for the future control of PCa.

Zinc finger E‐box binding homeobox 1 (ZEB1) is the critical epithelial–mesenchymal transition (EMT) activator and up‐regulates tumour cell plasticity and the EMT to acquire cancer stem cell properties.^12^ A recent study showed that ZEB1 repression by radiation enhanced lung adenocarcinoma cell migration and invasion capacity, as well as the EMT,^13^ while other previous studies showed that ZEB1 played an important role in the formation of vasculogenic mimicry (VM) in colorectal and breast cancers.^14^, ^15^ In PCa, ZEB1 was demonstrated to closely associate with EMT‐induced metastasis and stemness maintenance.^16^, ^17^ Moreover, the oncogene Src signalling was involved in the EMT and acquisition of cancer stem cell (CSC) phenotypes.^18^, ^19^, ^20^ For example, ZEB1 expression promoted lung cancer cell EMT through the activation of Fak/Src signalling,^21^ whereas the Src inhibition reduced the mammosphere formation and tumorigenesis potential of breast cancer stem cells.^18^ In addition, blockage of Src signalling compromised VM formation in malignant glioma cells.^22^ Accumulating evidence indicates that tumour EMT is relevant for the acquisition and maintenance of CSC characteristics and that it contributes to VM formation.^23^, ^24^

VM is a newly defined mechanism to supply nutrition to tumour cells and was described as the fluid‐conducting channel formed by highly aggressive tumour cells without endothelial cells.^25^ Tumour cells capable of VM formation have the commonality of a stem cell‐like, transendothelial phenotype after tumour tissues undergo hypoxia.^26^ VM formation has been linked to an unfavourable outcome of various human cancers.^27^, ^28^ In PCa, high levels of tumour tissue VM are associated with higher tumour Gleason score, TNM and metastasis.^29^ Thus, this study investigated the role and the potential contribution of ZEB1 in VM formation, EMT and CSC phenotypes in PCa tissues and the underlying molecular events in vitro. We hope to provide information regarding the role of ZEB1 in PCa development and progression.

## 2 MATERIALS AND METHODS

### 2.1 Patient samples

Tissue specimens were obtained from 96 PCa patients who underwent radical prostatectomy and transurethral resection prostate at The First Affiliated Hospital of Sun Yat‐sen University and Sun Yat‐sen University cancer centre between 2009 and 2013. All patients were diagnosed with prostate adenocarcinoma, and tissue specimens were obtained from surgery without any pre‐treatment. This study was approved by the Medical Ethics Committee of Sun Yat‐sen University, and informed consent was obtained from all patients before specimen collection.

### 2.2 Immunohistochemistry, haematoxylin and eosin staining and CD34/periodic acid–Schiff double staining

Immunohistochemical CD34/periodic acid–Schiff double staining was performed according to a previous study.^30^ A polyclonal rabbit anti‐ZEB1 antibody (1:100, Abcam, Cambridge, MA, USA), the EMT antibody sampler kit (1:200, Cell Signaling Technology, Danvers, MA, USA), Src antibody sampler kit (1:200, Cell Signaling Technology), polyclonal rabbit anti‐CD133 antibody (1:200, Proteintech, Rosemont, IL, USA), a goat anti‐rabbit IgG conjugated with horseradish peroxidase secondary antibody (1:5000, Good‐Science, Beijing, China) and mouse monoclonal IgG anti‐CD34 antibody (1:50, Zhongshan Goldenbridge, Beijing, China) were used for immunohistochemistry. The staining results were evaluated according to a previously described method.^31^

### 2.3 Cell lines and culture

Human PCa PC‐3 and LNCaP cell lines were purchased from the American Type Culture Collection (Manassas, VA, USA), while the DU‐145 cell line was a kind gift of Prof. Franky L. Chan (Faculty of Medicine, The Chinese University of Hong Kong, Hong Kong, China). PC‐3 and LNCaP cells were cultured in RPMI 1640 medium (Gibco BRL, Gaithersburg, MD, USA) supplemented with 10% foetal bovine serum (FBS; Gibco) and 1% penicillin/streptomycin (Gibco). DU‐145 cells were maintained in Dulbecco's modified Eagle's medium supplemented with 10% FBS and 1% penicillin/streptomycin at 37°C in a 5% CO_2_ incubator.

The Src inhibitor PP2 was purchased from MedChemExpress (Trenton, NJ, USA) and dissolved in dimethyl sulfoxide (DMSO) and then used to treat cells at concentrations between 0 and 20 μmol/L, while cells treated with DMSO alone were used as controls.^29^

### 2.4 Transient and stable transfections

The small interfering RNA kit was purchased from RiboBio (Guangzhou, China) and contained two efficient siRNA sequences targeting ZEB1 (siZEB1#1, 5′‐GGCAAGTGTTGGAGAATAA‐3′ and siZEB1#2, 5′‐CCAGAAATACACAGGGTTA‐3′). (pEnter‐ZEB1 and pEnter‐Src) plasmids were purchased from Vigene Bioscience (Jinan, China). These ZEB1 siRNA oligonucleotides and plasmid were transfected into PCa cells using Lipofectamine 2000^TM^ reagent (Invitrogen, Carlsbad, CA, USA) according to the manufacturer's instructions.

Lentiviral vector carrying green fluorescent protein (GFP) for ZEB1 knockdown was constructed by Jetway Biotech Co., Ltd. (Guangzhou, China). The shRNA sequences were the same as the siRNA sequences. The assay was performed as previously described.^32^

### 2.5 RNA isolation and qRT‐PCR

Total RNA was isolated from cells using an E.Z.N.A® HP total RNA kit (OMEGA, Norcross, GA, USA) and reversely transcribed into cDNA using a RevertAid first strand cDNA synthesis Kit (Thermo Scientific, Waltham, MA, USA). qPCR was performed with SYBR® Premix Ex Taq™ (TaKaRa, Tokyo, Japan) in a Mastercycler ep realplex PCR machine (Eppendorf, Hamburg, Germany) according to the kit instructions. Primer sequences used for qPCR are listed in Table SI. Relative mRNA levels of each gene were analysed in each sample using the 2^−ΔΔCt^ method against GAPDH mRNA.

### 2.6 Western blot

Protein extraction and Western blot were performed according to a previous study 29 with the following antibodies: a rabbit antibody against ZEB1 (1:1000, Abcam, Cambridge, MA, USA), the EMT antibody sampler kit (1:1000, Cell Signaling Technology, Danvers, MA, USA), the Src antibody sampler kit (1:1000, Cell Signaling Technology), a rabbit polyclonal anti‐CD133 antibody (1:1000, Proteintech, Rosemont, IL, USA), a mouse monoclonal anti‐GAPDH antibody (1:4000, Cwbiotech, Beijing, China) and a goat anti‐rabbit IgG conjugated with horseradish peroxidase (1:5000, Good‐Science, Shanghai, China). Visualization of protein bands was performed using the *Tanon*‐*5200* system (Biotanon, Shanghai, China).

### 2.7 Tumour cell three‐dimensional culture

This assay was performed to assess the capacity of tumour cells to form VM as described previously.^29^ Briefly, we first coated 96‐well plates with growth factor‐reduced Matrigel (BD Biosciences, Bedford, MA) at 50 μL/well. We then seeded tumour cells at a density of 4 × 10^4^ cells per well and incubated them at 37°C for 4 hours. After that, we counted the number of tube‐like structures in three randomly selected microscopic fields. The data were expressed as the mean ± SD for data analysis.

### 2.8 Wound‐healing assay

Cells were seeded in a six‐well plate and transfected with ZEB1 siRNA or plasmid for 48 hours. When the cells reached approximately 95% confluence, scratch wounds were made across the monolayer cells using a 200 μL pipette tip as described previously.^33^ After washed with PBS, the cells were further cultured in a complete growth medium for up to 48 hours, and the wound healing was photographed at various time‐points under an inverted microscope (Olympus, Tokyo, Japan) for three randomly selected sites per well.

### 2.9 Tumour cell invasion assay

Tumour cell invasion capacity was assessed using Transwell cell culture inserts with 8‐μm membrane pores that were pre‐coated with Matrigel (BD Biosciences, Bedford, MA, USA) and performed as described previously.^14^ The experiment was performed in triplicate and repeated at least once.

### 2.10 Colony formation assay

Tumour cell clonogenic ability was assessed using a colony formation assay as described previously with minor revisions.^34^ In brief, PCa cells were transiently transfected with ZEB1 siRNA or plasmid and then seeded in six‐well plates at a density of 500 cells per well and cultured for 15 days. Colonies were then fixed in 70% ethanol and stained with 0.5% crystal violet. Colonies with 50 cells or more were counted under an inverted microscope, and the data were expressed as the mean ± SD of three independent experiments.

### 2.11 In vivo tumour xenograft assay

This study was approved by the Institutional Animal Care and Use Committee (IACUC) of The First Affiliated Hospital, Sun Yat‐sen University (Guangzhou, China). Specifically, 12 male 6‐week‐old BALB/c nude mice were purchased from Nanjing Biomedical Research institute of Nanjing University (Nanjing, China) and maintained in a specific pathogen‐free (SPF) “barrier” facility and housed under controlled temperature and humidity and alternating 12‐hour light and dark cycles. The mice will receive SPF mouse chow and be allowed to drink sterile water ad libitum. For the assay, we firstly generated a stable ZEB1‐silenced PC3 cell subline; the mice were then randomly divided into two groups, that is an shControl group and shZEB1 group and subcutaneously injected with 5 × 10^6^ cells in 100 μL volume into the right armpit. Tumour growth was monitored and recorded every 7 days for 28 days with calliper. The tumour volume was calculated using the following formula: volume = (length [mm] × width^2^ [mm])/2. Four weeks later, mice were killed and tumour cell xenograft samples were resected and fixed in 10% buffered formalin for further experiments.

### 2.12 Statistical analysis

All statistical analyses were performed using SPSS 17.0 software (SPSS, Chicago, IL, USA). Depending on data sets, Student's *t* test, the chi‐square test, Fisher's exact test and Spearman correlation analysis were applied to evaluate the significant associations among categorical variables. A *P* < .05 was considered statistically significant.

## 3 RESULTS

### 3.1 Differential VM formulation and ZEB1 expression in PCa tissues

**Figure 1:**
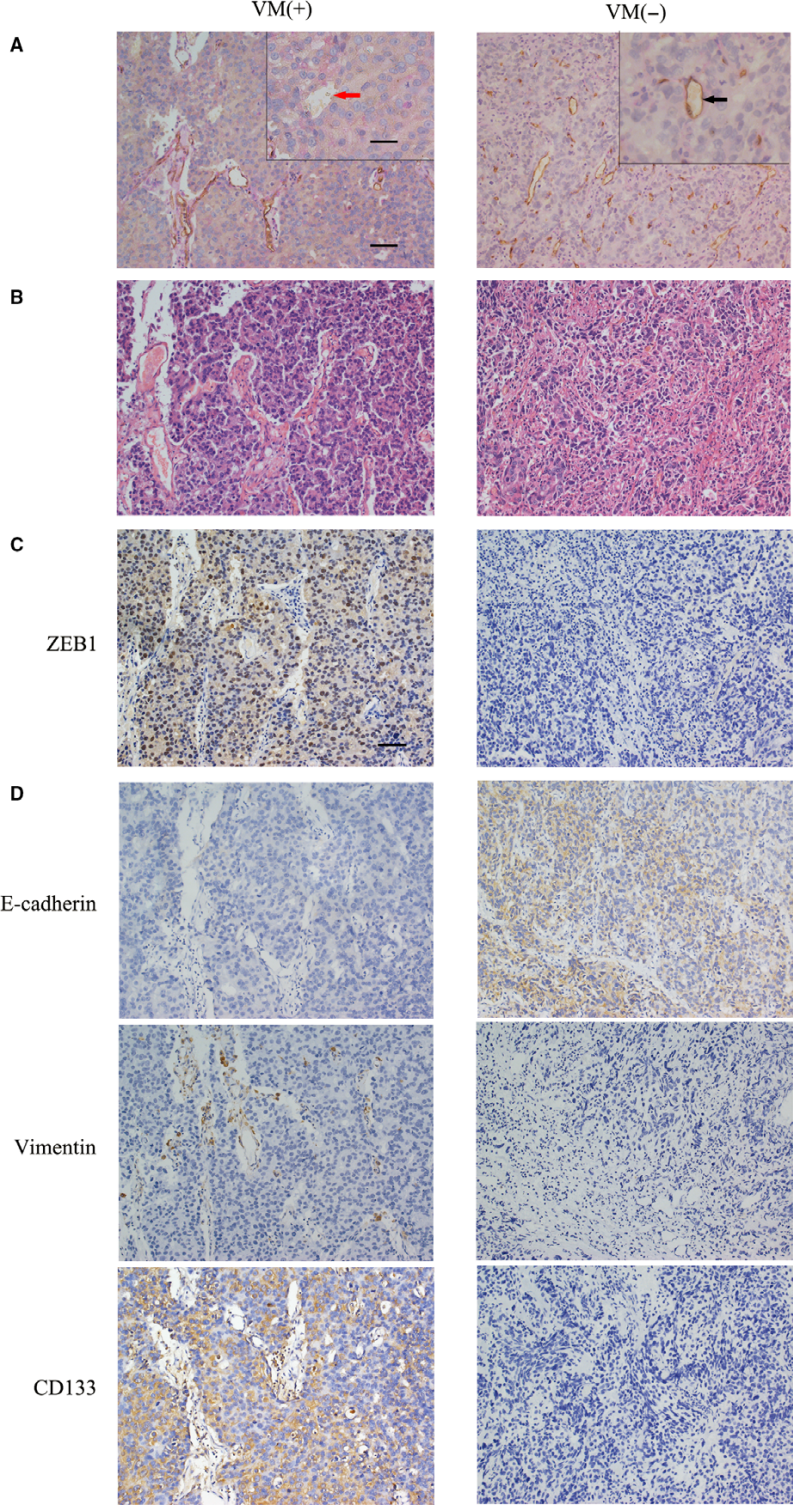
Immunohistochemistry, haematoxylin and eosin staining and immunohistochemical CD34/periodic acid–Schiff double staining. Paraffin‐embedded prostate cancer tissue specimens were double stained with periodic acid–Schiff stain, haematoxylin and eosin staining or immunostained. A, Identification of VM with immunohistochemical CD34 and periodic acid–Schiff double staining. Please refer to the method section for the criteria to identify VM; that is, a channel lined by tumour cells without CD34 staining is considered VM. This structure is indicated by a red arrow on the left panel (magnified in inset). The endothelium‐dependent vessel (indicated by a black arrow) that indicates positive CD34 staining is presented on the right panel. Note that red blood cells can be observed in the lumens of both VM and endothelium‐dependent vessels (magnified in inset). B, Haematoxylin and eosin staining. VM channel was surrounded by tumour cells. C, Immunohistochemistry. ZEB1 is strongly expressed in the nuclei or cytoplasm, and the expression of ZEB1 was higher in the VM‐positive sample (left) than the VM‐negative samples (right). D, The expression of EMT‐related proteins (E‐cadherin, vimentin) and the CSC‐associated protein CD133 in prostate cancer tissue specimens. E‐cadherin is strongly expressed on the cell membrane, and vimentin is primarily located in the extracellular matrix while CD133 is primarily cytoplasmic staining (Original magnification, ×200; scale bar, 20 μm, for insets, 10 μm)

In this study, we first assessed VM levels in PCa tissue specimens following our previous study.^29^ Tissue specimens underwent immunohistochemical CD34/periodic acid–Schiff double staining and haematoxylin and eosin staining to identify VM structures, which are defined as (1). the PAS‐positive or PAS‐negative loops with red blood cells, (2). they are negative for the endothelial cell marker CD34 immunostaining, and (3). they are surrounded by tumour cells. However, the blood vessels are formed by the endothelia that are positive for CD34 immunostaining (Figure 1A and B). As summarized in Table 1, VM was detected in 20 (20.8%) of 96 PCa specimens, and the presence of VM was significantly associated with higher Gleason score, TNM stage, and lymph node and distant metastases, but not with the patient's age (Table 1). For example, VM was significantly higher in PCa with a Gleason score of ≥8 (43.3%, 13/30) compared with a Gleason score of ≤7 (10.6%, 7/66). VM was more prevalent in PCa with ≥T3 stage (41.1%, 14/34) than that with ≤T2 stage (9.6%, 6/62).

**Table 1:** Association between VM level and ZEB1 expression with clinicopathological data from prostate cancer patients (n = 96)

Variables	n	VM level		*P* a	ZEB1 expression		*P*
		+	−		High (staining index ≥3)	Low (staining index <3)	
Age, years							
<66	44	11	33	.355	24	20	.52
≥66	52	9	43		25	27	
Gleason score							
≤7	66	7	59	.000	28	38	.012
≥8	30	13	17		21	9	
T classification							
≤T2	62	6	56	.000	25	37	.005
≥T3	34	14	20		24	10	
Lymph node metastasis							
No	83	12	71	.000	38	45	.021
Yes	13	8	5		11	2	
Distant metastasis							
No	89	16	73	.048	42	47	.012
Yes	7	4	3		7	0	

a Analysed by chi‐square (χ^2^) test.

We then immunostained ZEB1 protein in the duplicated PCa tissue sections and scored high vs low expression of ZEB1 protein in these samples. To compare VM positivity, we divided these tissue samples into two groups. As shown in Figure 1C, a high level of ZEB1 expression was presented in 47 of the 92 cases (51.0%) vs low ZEB1 expression in 49 cases (49.0%). The high level of ZEB1 protein was strongly associated with the presence of VM, that is 16 of these 20 (80%) samples with positive VM overexpressed ZEB1 protein vs only 33 of the 76 (43.3%) samples with negative VM expressed ZEB1 protein (*P* = .008; Table 2). The expression of ZEB1 protein was also associated with higher Gleason score, TNM stage, and lymph node and distant metastases (Table 1). These data indicated that ZEB1 could regulate tumour VM formation, invasion and metastasis.

**Table 2:** Association between VM and ZEB1 expression in prostate cancer tissues

VM	ZEB1 expression		*P* value^a^
	High	Low	
+ (20)	16	4	.008
− (76)	33	43	

a Analysed by chi‐square (χ^2^) test.

### 3.2 VM association with the expression of EMT/CSC‐related proteins in PCa tissues

We then associated VM formation with tumour cell EMT and cancer stem cell phenotypes in PCa consecutive tissue sections. In Figure 1D, we found that VM‐positive specimens were more likely to express a high level of vimentin and CD133 protein but lacked E‐cadherin expression. Notably, there was a significant association between the expression of EMT markers (E‐cadherin vs vimentin) and the presence of VM (*P* = .045 and *P* = .036, respectively; Table 3) in PCa tissues. Similarly, the presence of VM was also associated with the expression of a CSC marker, CD133 (*P* = .003).

**Table 3:** Association between VM and ZEB1 expression with different protein expressions in prostate cancer tissues

Variables	VM		*P* a	ZEB1		*r*
	+	−		High	Low	
E‐cadherin						
High	5	38	.045	13	30	−0.375
Low	15	38		36	17	
Vimentin						
High	12	26	.036	28	10	0.367
Low	8	50		21	37	
CD133						
High	13	22	.003	29	6	0.482
Low	7	54		20	41	

a Analysed by chi‐square (χ^2^) test.

### 3.3 ZEB1 association with the expression of EMT/CSC‐related proteins in PCa tissues

In Figure 1D, we also found that ZEB1‐expressed PCa cells had down‐regulated E‐cadherin expression, which was an inverse association (*r* = −0.375; Table 3). However, ZEB1 expression was demonstrated to associate with vimentin and CD133 expression (*r* = 0.367 and *r* = 0.482, respectively; Table 3). Taken together, there could be interplay between ZEB1, EMT/CSC and VM formation.

### 3.4 ZEB1 regulated VM formation and expression of EMT‐related and CSC‐associated proteins

**Figure 2:**
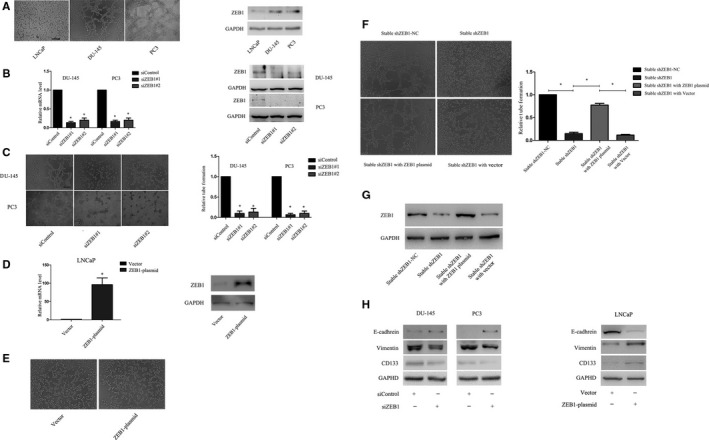
Association between ZEB1 expression and VM formation in prostate cancer cell lines. ZEB1 expressions were assayed using Western blot in prostate cancer cell lines, while VM formation was assessed using tumour cell three‐dimensional culture. A, Association between ZEB1 expression and VM formation in prostate cancer cell lines. PC3 and DU‐145 cells expressed high levels of ZEB1 protein and were able to form tubular structures in culture, whereas LNCaP cells expressed a low level of ZEB1 protein and were not able to do so. B, Messenger RNA and protein levels of ZEB1 were significantly decreased 48 hours after transfection. C, The knockdown of ZEB1 expression significantly reduced the number of tubular structures formed by PC3 or DU‐145 cells (Original magnification, ×100; scale bar, 40 μm). D, Levels of ZEB1 mRNA and protein were significantly increased by ZEB1 cDNA transfection in LNCaP cells. E, The overexpression of ZEB1 in LNCaP cells incapable of forming tubular structures. F, Rescue experiment. The shZEB1 PC3 cells reconstructed tubular structures resulted from rescue of ZEB1 expression. G, Western blot showed that expression of ZEB1 was fully restored in shZEB1 PC3 cells after transfected into ZEB1 plasmid. H, Western blot showed that the depletion of ZEB1 expression in prostate cancer cell lines resulted in decreased VE‐cadherin and CD133 levels but an increase in the E‐cadherin level, and all these were reversed by ZEB1 up‐regulation

To assess and confirm the role of ZEB1 in VM formation, we first measured ZEB1 expression in PCa cell lines (PC3, DU‐145 and LNCaP). Similar to the result of our previous study,^29^ we found that androgen‐independent PC3 and DU‐145 cells could form typical vessel‐like tubes in the three‐dimensional culture but that androgen‐dependent LNCaP cells could not (Figure 2A). We also found that LNCaP expressed the lowest level of ZEB1 protein as that by PC3 and DU‐145 (Figure 2A). We then knocked down ZEB1 expression in PC3 and DU‐145 cells using siRNA, whereas overexpressed ZEB1 expression in LNCaP cells was performed using plasmid. As shown in Figure 2B and C, ZEB1 expression was significantly reduced after ZEB1 siRNA transfection, and the number of tubular structures was also remarkably decreased. However, LNCaP cells unexpectedly failed to form tubular structures after ZEB1 overexpression (Figure 2D,E). Because overexpression of ZEB1 did not induce tubular structures in LNCaP cells, we chose stable ZEB1 knockdown PC3 cells to rescue its endogenous ZEB1 expression in order to eliminate siRNA off‐target effect in ZEB1‐interfered cells. As shown in Figure 2F and G, rescue of ZEB1 expression restored the VM behaviour in ZEB1 knockdown PC3 cells. Moreover, the knockdown of ZEB1 expression resulted in down‐regulations of vimentin and CD133 but the up‐regulation of E‐cadherin; these alternations were reversed by ZEB1 up‐regulation (Figure 2H).

### 3.5 Reduction of PCa cell migration, invasion and clonogenicity after ZEB1 knockdown

**Figure 3:**
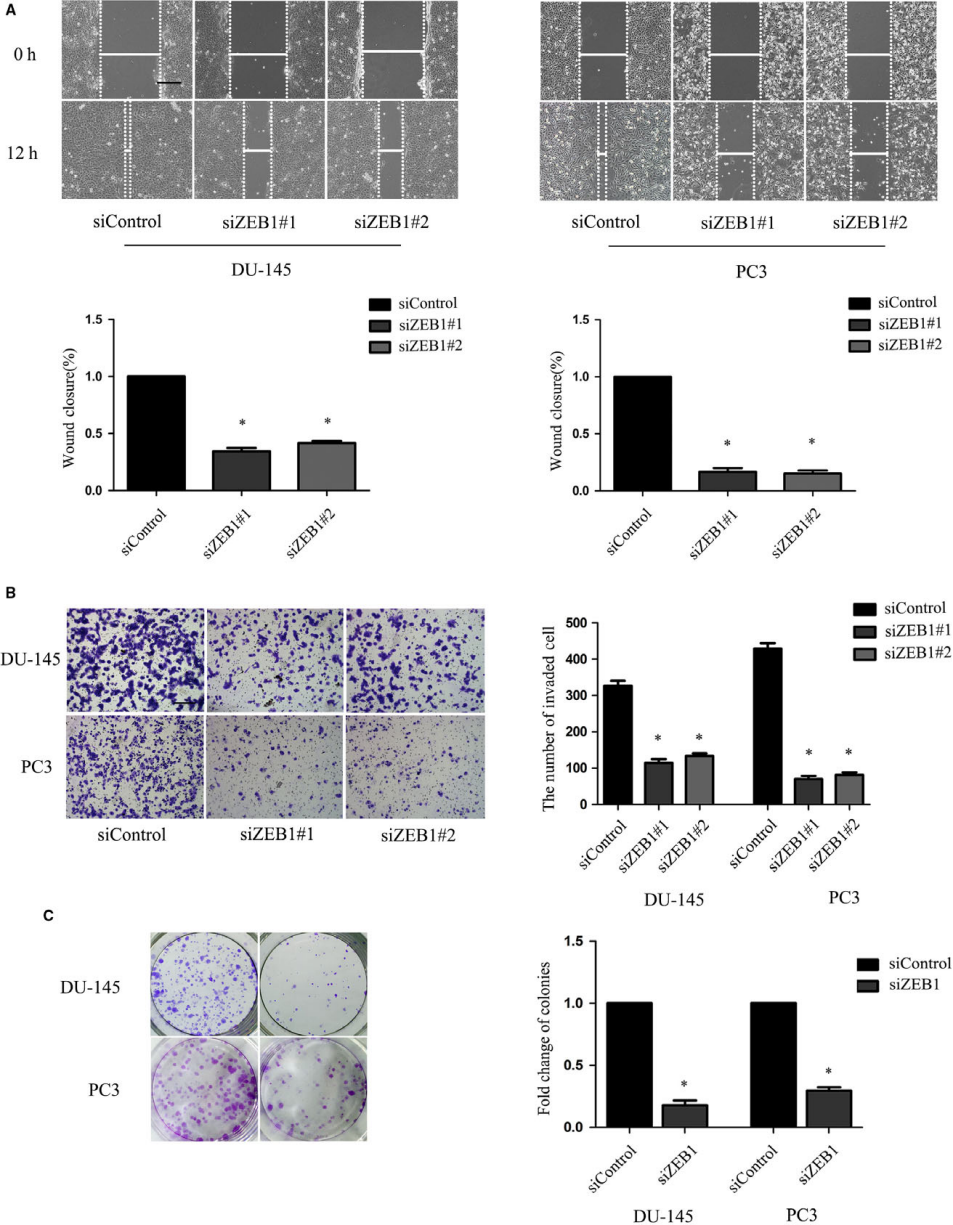
Effects of ZEB1 knockdown on the regulation of prostate cancer cell migration, invasion and clonogenicity. A, Wound‐healing assay. Tumour cell migration was significantly suppressed 12 h after the knockdown of ZEB1 expression. B, Transwell invasion assay. Compared with the negative control siRNA, ZEB1 siRNA dramatically decreased the number of cells invading through the Matrigel‐coated Transwell membranes (Original magnification, ×100; scale bar, 40 μm). C, Clone formation assay: tumour cells transfected with control oligos formatted larger and more colonies compared with tumour cells transfected with siZEB1

We then evaluated the effect of ZEB1 knockdown on the regulation of tumour cell migration and invasion capacity. The wound‐healing assay showed that ZEB1 knockdown significantly reduced PC3 and DU‐145 cells migration (Figure 3A), while the Transwell invasion assay showed that the down‐regulation of ZEB1 expression significantly down‐regulated tumour cell invasion capacity (Figure 3B). Similarly, ZEB1 knockdown significantly suppressed the colony formation of PC3 cells by 55.0% and DU‐145 cells by 73.3% (Figure 3C).

### 3.6 ZEB1 overexpression promoted PCa cell migration, invasion and clonogenicity abilities

**Figure 4:**
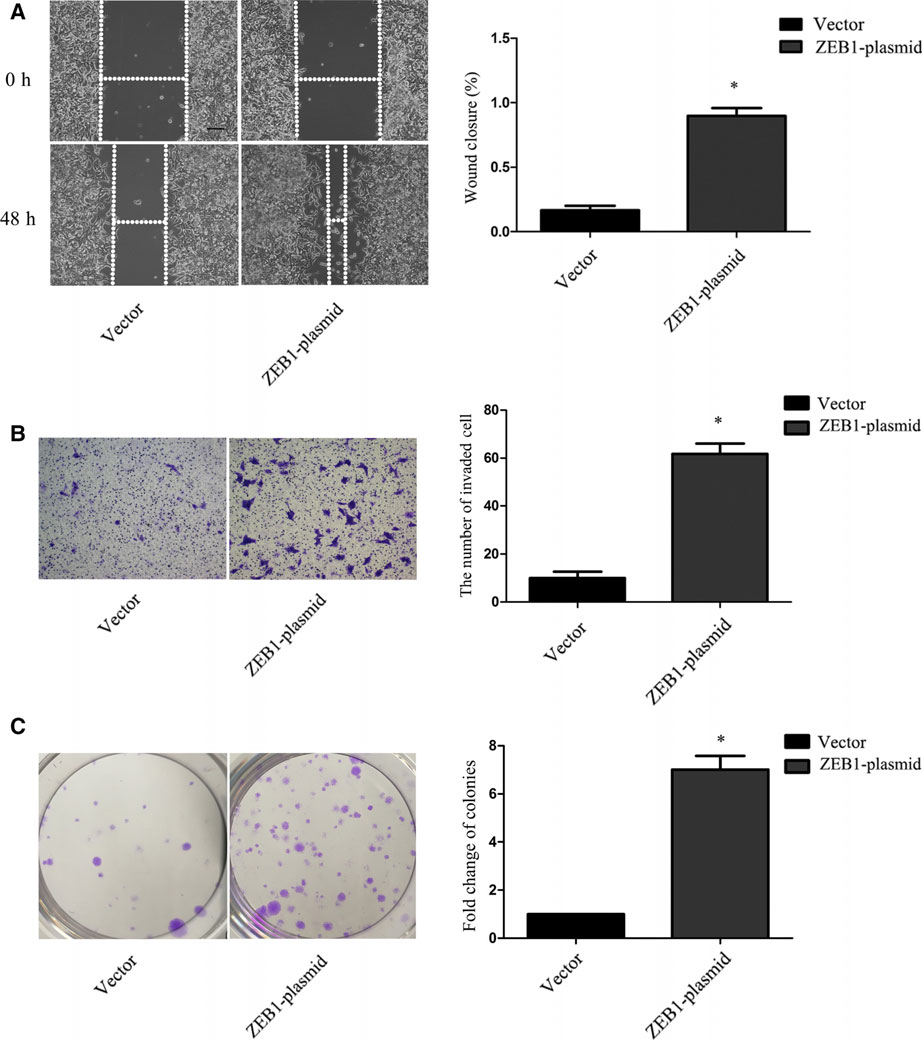
Changes in tumour cell migration, invasion and clonogenicity after ZEB1 cDNA transfection. A, Wound‐healing assay. LNCaP cell migration was significantly increased 48 h after ZEB1 overexpression. B, Transwell invasion assay. ZEB1 up‐regulation dramatically increased the number of cells invading through the Matrigel‐coated Transwell membranes compared with the negative control (Original magnification, ×100; scale bar, 40 μm). C, Clone formation assay. Tumour cells transfected with ZEB1 cDNA formed larger and more colonies compared with tumour cells transfected with control vector

To further confirm the role of ZEB1 in aggressive cancer phenotypes, we ectopically overexpressed ZEB1 in LNCaP cells. The wound‐healing assay demonstrated that ZEB1 overexpression significantly induced LNCaP cell migration (Figure 4A), while the Transwell invasion assay showed that the up‐regulated ZEB1 expression significantly enhanced tumour cell invasion capacity (Figure 4B). Furthermore, the overexpression of ZEB1 remarkably up‐regulated the clonogenic potential of LNCaP cells (Figure 4C).

### 3.7 Src signalling mediation of ZEB1‐induced VM formation and gene expression

**Figure 5:**
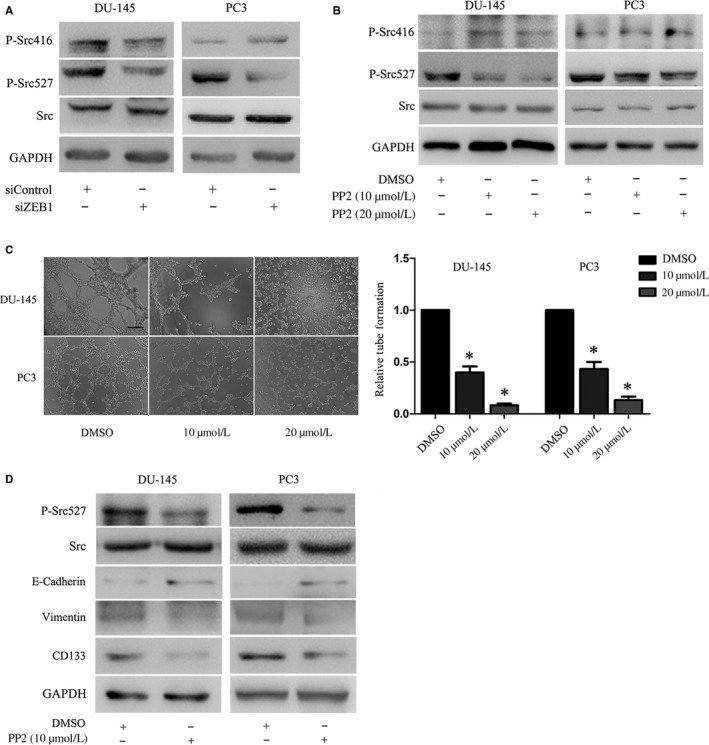
The Src signalling mediation of ZEB1‐induced VM formation and gene expression. A, Western blot. The knockdown of ZEB1 decreased the level of p‐Src527 in prostate cancer cell lines. The p‐Src426 level was decreased in DU‐145 but increased in PC3 cells. B, Western blot. Prostate cancer cells were grown and treated with different concentrations of the Src inhibitor PP2 for 2 h and subjected to Western blot analysis. The data show that the inhibition of Src dose dependently decreased levels of p‐Src527 but not p‐Src426. C, Tumour cell 3‐D culture. The similarly treated tumour cells were assayed in the 3‐D culture. The number of tubular structures formed by PC3 and DU‐145 cells was decreased in a dose‐dependent manner after treatment with various concentrations of PP2 (0‐20 μmol/L) (original magnification, ×100; scale bar, 40 μm). D, Prostate cancer cells were grown and treated with 10 μmol/L of the Src inhibitor PP2 for 2 h and subjected to Western blot analysis. The down‐regulation of ZEB1 led to decreasing expressions of VE‐cadherin and CD133 but restored expression of E‐cadherin

**Figure 6:**
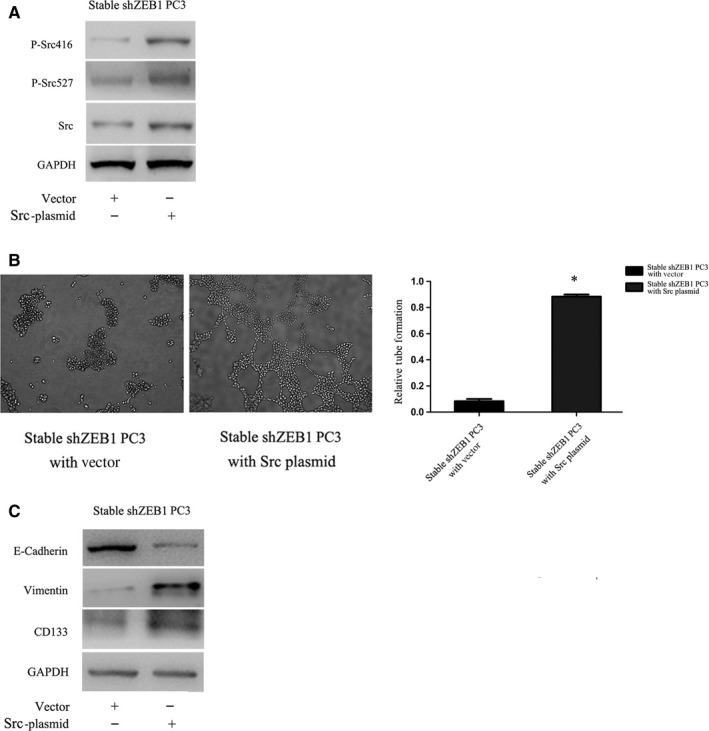
Src overexpression restored the VM phenotype and gene expression in ZEB1‐silenced PC3 cells. A, Expression levels of Src, p‐Src527 and p‐Src426 were significantly elevated followed by Src cDNA transfection. B, Tubular structures reoccurred after the rescue of Src in ZEB1‐silenced PC3 cells. C, Rescue of Src led to an increasing expression of VE‐cadherin and CD133 but decreasing E‐cadherin expression

ZEB1 and Src kinase were shown to modulate PCa cell metastatic phenotypes.^35^ Hence, we investigated whether Src contributed to ZEB1‐dependent VM formation and found that ZEB1 knockdown down‐regulated the level of p‐Src^527^ in both PC3 and DU‐145 cell lines but dramatically enhanced the level of p‐Src^416^ in PC3 and decreased level of p‐Src^416^ in DU‐145 (Figure 5A). We thus assessed the role of Src signalling using PP2, a specific inhibitor of Src signalling. As shown in Figure 5B, we observed that PP2 dose dependently reduced the p‐Src^527^ level but not the p‐Src^416^ level in PC3 and DU‐145 cells. In parallel, the tubular structures gradually disappeared following PP2 treatment in a dose‐dependent manner (Figure 5C). In addition, the expression of vimentin and CD133 was also partially reduced, whereas E‐cadherin expression was increased after treatment of these PCa cell lines with 10 μmol/L PP2 (Figure 5D). The data indicated that phosphorylation at Tyr‐527 of Src signalling was required for VM formation and gene expression. Hence, we next transiently transfected the Src plasmid into stable ZEB1 knockdown cells and observed that the overexpression of Src restored VM formation (Figure 6A and B). As presented in Figure 6C, similarly, a reoccurrence of VM behaviour was accompanied with the up‐regulation of vimentin and CD133 and down‐regulation of E‐cadherin. Taken together, these data indicated that Src signalling mediated ZEB1‐induced VM formation and gene expression and may act by activating Tyr‐527.

### 3.8 Depletion of ZEB1 restrained tumour growth and VM formation in vivo

**Figure 7:**
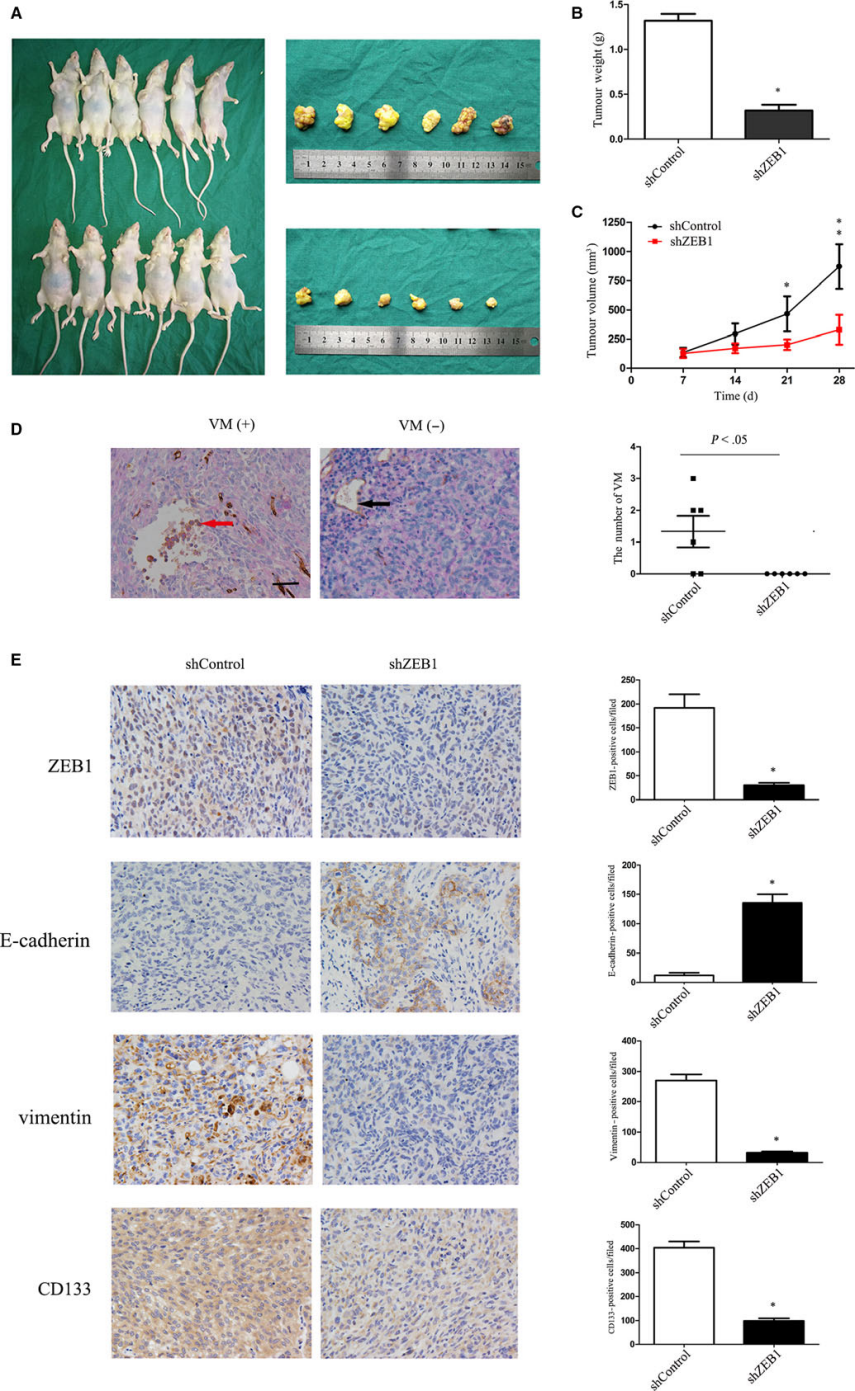
Effects of siZEB1‐transfection on regulation of tumour growth and VM formation in prostate cancer cell xenografts. A,B, Volume of shZEB1 cell xenografts was significantly smaller than that of shControl cell xenografts. C, Tumour volumes were measured every 7 days (*P* < .01). Stable knockdown of ZEB1 induced growth inhibition of xenografts. D, CD34 and PAS double staining identified VM channel in xenografts. The VM channels were PAS positive but CD34 negative (red arrow). The endothelium‐dependent blood vessel was CD34 positive (black arrow). E, Differential expression of ZEB1, E‐cadherin, vimentin and CD133 in the indicated groups (magnification, ×400; scale bar, 10 μm)

To further confirmed the effect of ZEB1 on PCa VM, shControl or shZEB1 PC3 cells were subcutaneously injecting into nude mice. We observed that average tumour volume was significantly decreased in shZEB1 group in comparison with the shControl (*P* < .05, Figure 7A and B). Moreover, tumour xenografts of the shZEB1 group were growing more slowly than those of the shControl group (*P* < .05, Figure 7C).

Immunohistochemical analysis of tumour cell xenografts with the CD34/PAS double staining showed that VM was more prevalent in the shControl (4/6) than that in the shZEB1 group (0/6, *P* < .05, Figure 7D). Compared with the corresponding control, expression of vimentin and CD133 was also decreased, whereas E‐cadherin expression was increased in the shZEB1 group (Figure 7E).

## 4 DISCUSSION

In this study, we firstly demonstrated an association between ZEB1 expression and VM formation in PCa tissue specimens. We then associated VM formation and ZEB1 expression with higher Gleason score, TNM stage, and lymph node and distant metastases, as well as with the expression of vimentin and CD133. In vitro, ZEB1 was required for VM formation and mediated the expression of EMT‐related and CSC‐associated proteins in PCa cells. ZEB1 also facilitated PCa cell migration, invasion and colony formation. Similar result was also shown in our in vivo study and depletion of ZEB1 protein in PC3 cells restrained growth of tumour cell xenograft in nude mice. Furthermore, Src signalling was essential for ZEB1‐induced VM formation and gene expression through p‐Src^527^ activation. These data suggest that ZEB1 may serve as a novel therapy inhibiting VM formation that may therefore effectively control VM‐positive PCa progression.

Notably, VM is a known novel vascular network pattern that is formed by tumour cells but not endothelial cells in various cancers, including breast cancer, glioma and PCa.^25^, ^29^, ^36^, ^37^ The VM structure was firstly reported by Maniotis et al,^25^, ^29^, ^36^, ^37^ and the VM is formed by aggressive tumour cells gaining the characteristics of transdifferentiation and acquiring endothelial cell behaviour to access nutrition and initiate metastasis.^38^, ^39^ A previous systematic review with a meta‐analysis showed that tumour lesions with VM structures had worse 5‐year survival, indicating that VM was a predictor of poor prognosis in multiple cancers.^40^ In the current study, we further confirmed our previous data showing that VM was significantly associated with higher Gleason score, advanced TNM stages and tumour metastasis.^29^

We then explored the molecular mechanism underlying VM formation by investigating the association between ZEB1 expression and VM. As a transcription factor, ZEB1 is a well‐known EMT inducer that plays a vital role in tumour initiation, tumour cell plasticity and the acquisition of stemness.^12^, ^41^ A previous study demonstrated that ZEB1 expression enhanced the aggressive tumour cell phenotype by remodelling lung cancer extracellular matrix.^42^ ZEB1 expression also promoted tumour initiation and progression, which resulted from inhibiting the senescence of colorectal cancer cells.^43^ The current study demonstrated that ZEB1 was significantly correlated with Gleason score, TNM and metastasis, in a manner similar to VM. Our further experiments showed that the epigenetic silencing of ZEB1 expression abrogated the ability of tumour cells to form tubular structures, while rescued ZEB1 expression restored the tubular structures in vitro or in vivo. However, ectopic ZEB1 expression in LNCaP cells did not induce VM formation, indicating that ZEB1 was necessary but not sufficient for VM formation, and a certain required factor remains yet to be determined. Nevertheless, our current finding demonstrated that ZEB1 played a role in regulating VM formation in PCa vivo and in vitro and provided an explanation for ZEB1 promotion in cancer progression.

Furthermore, the current study showed that both VM and ZEB1 expression were associated with the expression of EMT‐related and CSC‐associated proteins in PCa cells and tissues. Notably, VM formation represented a remarkable example of cell plasticity.^44^ The tumour cell EMT refers to cancer cells losing epithelial features but acquiring a mesenchymal phenotype, which is also known as a phenomenon of cell plasticity.^45^ A recent study showed that tumour cells underwent the EMT by gaining stem cell‐like properties and that plasticity of the subset of cancer cells could mimic the pattern of embryonic vasculogenesis in a structure similar to VM.^46^, ^47^, ^48^ Thus, our current study further confirmed the interplay between ZEB1, VM and EMT‐related and CSC‐associated proteins in vitro and in vivo. However, further gene knock‐in and knockout experiments are needed to verify their interaction and molecular signalling.

In addition, the current study further explored the role of ZEB1 in VM formation in PCa cells. The Src kinase is a non‐receptor tyrosine kinase and was involved in regulating malignant behaviours in tumour cells,^49^, ^50^ whereas recently, Fak/Src signalling was shown to mediate the effects of ZEB1‐induced extracellular matrix degradation and in turn to enhance lung cancer invasion and metastasis.^21^ Thus, the current study assessed whether the Src signalling regulated ZEB1‐induced VM formation. Our data showed that ZEB1 knockdown reduced VM formation in parallel with the inhibition of Src phosphorylation in the p‐Src^527^ site in PCa cells. We further confirmed that the treatment of PCa cells with the Src inhibitor PP2 resulted in a reduction in VM formation, whereas Src overexpression in stable ZEB1 knocked down cells could restore VM formation. Of note, PP2 inhibited the level of p‐Src^527^, which was coincident after the knockdown of ZEB1 expression in PCa cells. Although the PP2‐reduced p‐Src^527^ level contradicted that of a previous study showing that p‐Src^416^ was taken as active and p‐Src^527^ as inactive forms of the Src enzyme, our current data are consistent with a previous study.^50^, ^51^ Thus, further study is needed to address this discrepancy. These data may indirectly indicate that the phosphorylation of the p‐Src^527^ site was able to functionally activate Src. Furthermore, Src was not only required to maintain cancer stem cell properties but also participated in the pathway that controls the EMT.^52^, ^53^ Unsurprisingly, the expression of vimentin and CD133 was decreased while the expression of E‐cadherin was increased after the inhibition of Src. Taken together, our data identify the role of Src signalling in ZEB1‐dependent EMT and CSC properties as well as their role in VM formation.

## 5 CONCLUSIONS

Our current study was the first to reveal that ZEB1 played an important role in PCa VM formation in vivo and in vitro. Mechanistically, Src signalling mediated the effects of ZEB1 in PCa cells. Thus, this study provided a novel insight into the molecular mechanism of VM formation and may be used as novel therapeutic targets in controlling VM‐positive PCa.

## CONFLICT OF INTERESTS

All authors declared there was no conflict of interests involved in this study.
