# Supplementary material for: ZEB1‐mediated vasculogenic mimicry formation associates with epithelial–mesenchymal transition and cancer stem cell phenotypes in prostate cancer
Source: J Cell Mol Med. 2018 May 12;22(8):3768–81. doi: 10.1111/jcmm.13637 (PMC6050489; doi:10.1111/jcmm.13637)
Supplement: Supplementary file 1 [file JCMM-22-3768-s001.docx]

Table SⅠ. Primer sequences used in qPCR

| Gene name | primers | Tm (°C) |
| --- | --- | --- |
| ZEB1 | 5'-GATGATGAATGCGAGTCAGATGC-3' | 60.12 |
|  | 5'-CTGGTCCTCTTCAGGTGCC-3' | 59.70 |
| E-cadherin | 5'- AGTGTCCCCCGGTATCTTCC-3' | 60.69 |
|  | 5'-CAGCCGCTTTCAGATTTTCAT-3' | 57.50 |
| Vimentin | 5'- CCCTCACCTGTGAAGTGGAT -3' | 59.01 |
|  | 5'- TCCAGCAGCTTCCTGTAGGT -3' | 60.55 |
| CD133 | 5'-TTGCGGTAAAACTGGCTAAG-3' | 56.35 |
|  | 5'-TGGGCTTGTCATAACAGGAT-3' | 56.50 |
| GAPDH | 5'-CGACCACTTTGTCAAGCTCA-3' | 58.42 |
|  | 5'-AGGGGAGATTCAGTGTGGTG-3' | 59.01 |
